# New Appliances and Things Medical

**Published:** 1895-11-30

**Authors:** 


### NEW APPLIANCES AND THINGS MEDICAL.

### PEAT WOOL AS A SURGICAL DRESSING.

The marvellous powers of absorbence and resistance to putrefaction possessed by certain preparations of peat have been recognised for a good many years past in various parts of Europe, notably perhaps in Germany and Sweden, where in the form of a coarse powder this substance has been proved of the utmost value in connection with the dry system of sewage removal. Further investigation into its special properties led many eminent German surgeons to advocate its use as a surgical dressing at the Surgical Congress in 1884, Dr. Leisrink drawing particular attention to its applicability for army surgery. Great improvements have been effected in the preparation of the wool for this purpose during recent years, and its increasing use as a dressing only serves to confirm the very favourable opinion formed by those who first had experience of its value. Peat itself is naturally divided into two layers, the upper or "moss peat" and the lower or "black peat"; it is from the fibre of the former, the light moss peat, that the soft absorbent wool for surgical purposes is prepared. Microscopic examination of the cell structure reveals the reason for its peculiar properties. The cells are provided with openings in their outer walls, and consequently suck in liquids, instead of taking them up by capillary attraction, as in the case of ordinary absorbent dressings. This being so it follows that wound discharges thus absorbed into the interior of the cells are excluded from contact with the air; and it is to this cause that must be attributed the absence of smell and decomposition where peat dressings are used, and the greater durability of the material, as well as to the fact that peat has been proved to contain some substance which does, as stated by a writer on the subject in the *Lancet* of July 21st, 1894, "definitely retard the development of micro-organisms." The same writer states that microscopically he found the peat fibres to consist "almost entirely of very long and very narrow cells with finely tapering ends. The fine peat fibres consisted of bundles of from fifty to one hundred of these cells, which were from one-fifth to one-quarter the diameter of fine cotton fibres. They were perfectly homogeneous and glassy; they had a minute cell cavity which appeared as a fine dark line, with no trace whatever of stomata. Besides these every here and there was a much broader cell with transverse marking, resembling the scalariform vessels of plants, and there were a few polygonal vegetable cells adherent to these. The most striking points were the small size of the fibres, their great length, transparent, homogeneous structure, and regular grouping." Its great cheapness, combined with its aseptic advantages, makes peat dressing a most important adjunct to modern surgery. It is evident that for use in time of war it is simply invaluable, and this fact has been so recognised in France that the War authorities have there adopted it in the military hospitals in place of the wools and gauzes hitherto in use, and was largely used in the recent expedition to Madagascar. In England it is only just beginning to be at all generally known. The Peat Industries Syndicate (Limited), 32, Queen Victoria Street, E.C., showed specimens of the various preparations at this year's British Medical Association meeting, where it met with considerable approval, and every variety of the dressing can be inspected at the Queen Victoria Street offices. Two qualities are kept in stock, and made up as desired in sheets or pads, enclosed in gauze or otherwise, and in rolled packets of one pound and half-pound. Whether it will become popular for hospital use remains to be seen. Its lower cost is greatly in its favour, but the slight disadvantage of its brown colour and a propensity to make a little dust when handled may militate there against its general adoption. Its very great value as a surgical dressing is beyond all question.

